# Updating “A dataset on patient-individual lymph node involvement in oropharyngeal squamous cell carcinoma” with an additional dataset from a second institution

**DOI:** 10.1016/j.dib.2025.111546

**Published:** 2025-04-10

**Authors:** Sergi Benavente, Roman Ludwig, Panagiotis Balermpas, Jan Unkelbach

**Affiliations:** a Departement of Radiation Oncology, University Hospital Zurich, Rämistrasse 100, 8091 Zurich (CH), Switzerland; b Department of Radiation Oncology, Vall d'Hebron University Hospital, Pg. de la Vall d'Hebron 119, 08035 Barcelona (ES), Spain

**Keywords:** Head & neck squamous cell carcinoma, Oropharynx, Patterns of progression, Lymphatic involvement, Graphical user interface

## Abstract

With this update, we add 164 patients with newly diagnosed oropharyngeal squamous cell carcinoma (OPSCC) from the University Hospital Vall d'Hebron (HVH) in Barcelona, Spain, to the previously published cohort of 287 OPSCC patients from the University Hospital Zurich (USZ). For each patient, we report the clinical involvement of lymph node levels (LNLs) I-V and VII on both sides of the neck. LNL involvement is assessed separately for the available diagnostic modalities comprising computed tomography (CT), magnetic resonance imaging (MRI), and/or ^18^FDG-positron emission tomography (PET/CT). For 10 surgically treated patients, we also report pathological LNL involvement after neck dissection. Additionally, we report clinicopathological factors such as sex, age, alcohol and nicotine abuse, HPV status, TNM stage, tumor subsite (ICD-10 code) and tumor volume, and whether the tumor extended over the mid-sagittal plane.

The additional data is made available in the same CSV file format as the records of the initial dataset. The new data represents a valuable update to the original records that substantially increases the size of the cohort. In addition, it allows assessing differences between datasets, which provides information on potential patient biases. Due to the same data format, it is straightforward to reproduce any analysis that was done on the original data with the extended dataset.

Specifications Table

**Table:** 

Subject	Oncology
Specific subject area	Quantification of lymphatic metastatic progression in oropharyngeal squamous cell carcinoma
Type of data	Table
Data collection	Patients treated at the HVH between 2004 and 2021 with newly diagnosed OPSCC are included. The data was acquired during routine clinical care and retrospectively extracted by an experienced radiation oncologist. Essentially, we used the same methodology as in the original article. Data was extracted from electronic medical records and from radiological images such as computed tomography (CT), magnetic resonance (MR), and positron emission tomography (PET).
Data source location	Institution: University Hospital Vall d'Hebron City/Town/Region: Barcelona Country: Spain Latitude and longitude: 41.42837965756258, 2.1412449620632317
Data accessibility	Repository name: GitHub, rmnldwg/lydata Direct URL to data: https://github.com/rmnldwg/lydata Folder/File: 2025-hvh-oropharynx/data.csv Data identification number: https://doi.org/10.5281/zenodo.14976454 The dataset can also be downloaded from our web-based GUI: https://lyprox.org
Related data article	Ludwig, R. *et al.* A dataset on patient-individual lymph node involvement in oropharyngeal squamous cell carcinoma. *Data Br.***43**, 108345 (2022). [1]

## 1 Value of the Data

• The new data expands the already available data by a substantial number of patients from another institution. This reduces the statistical uncertainty in parameters estimated from the data, in this case the probabilities of OPSCC to spread to different LNLs. In addition, the differences between datasets provides information on potential patient selection biases.
• The data benefits researchers interested in quantifying the patterns of lymphatic tumor progression in head and neck cancer. Researchers may use the data to formulate hypotheses on the correlations between LNL involvement and clinicopathological factors. It may also be used to train statistical models predicting lymphatic tumor progression.

## 2 Background

Radiotherapy of HNSCC includes the elective irradiation of parts of the lymph drainage region that appear healthy on biomedical imaging but are at risk of harboring occult lymph node metastases. Current guidelines for selecting the regions to be irradiated rely mostly on the overall prevalence of lymph node metastases reported in each lymph node level (LNL) [[2], [3], [4], [5]–6]. However, the elective nodal clinical target volume (CTV-N) may be further personalized through a quantitative risk estimate for occult metastases that is based on a patient's individual clinical diagnosis [7,8]. This diagnosis encompasses clinicopathological factors such as tumor location, T-category, and the location of clinically detected metastases. Developing these quantitative models requires datasets like the one presented in this work. The larger the dataset, the smaller the statistical uncertainty in the model parameters estimated from the data.

## 3 Data Description

The repository at https://github.com/rmnldwg/lydata as well as the zenodo archive (https://doi.org/10.5281/zenodo.14976454) contain the directory `2025-hvh-oropharynx` with the following file tree:



The data is provided as a CSV-table through the file `data.csv`. It represents a curated and cleaned version of the file `raw.csv` containing the raw data as it was extracted. The `README.md` is a markdown formatted text file that contains information on the data. `CITATION.cff` is a text file that contains details on how to cite the data in this subfolder. It follows the specifications of the Citation File Format (CFF). Inside the `figures` subfolder, we provide images in the PNG format that plot basic characteristics of the patient cohort, also regarding the difference of this data to the USZ cohort.

The `data.csv` is provided as a CSV-table containing one row for each of the 164 patients. The table has a header with three levels (i.e., it spans the first three lines in the file) that describe the columns. Below, we explain each column in the form of a list with three levels:

1) **patient:** General information about the patient's condition can be found under this top-level header.
  1) **#:** The second level under patient has no meaning and exists solely as a filler.
    1) **id:** Enumeration of the patients
    2) **institution:** The clinic at which the patients were treated and recorded. This holds the value "Vall d'Hebron Barcelona Hospital" for all patients in this dataset.
    3) **sex:** Sex of the patient
    4) **age:** Patient's age at diagnosis
    5) **diagnose_date:** Date of diagnosis (format YYYY-mm-dd) defined as the date of first histological confirmation of HNSCC.
    6) **alcohol_abuse:** True for patients who stated that they consume alcohol regularly, false otherwise
    7) **nicotine_abuse:** True for patients who have been regular smokers (> 10 pack years)
    8) **hpv_status:** True for patients with human papilloma virus associated tumors (as defined by p16 immunohistochemistry)
    9) **neck_dissection:** Indicates whether the patient has received a neck dissection as part of the treatment.
    10) **tnm_edition:** The edition of the TNM classification used to classify the patient [9]
    11) **n_stage:** Degree of spread to regional lymph nodes
    12) **m_stage:** Presence of distant metastases

2) **tumor:** Information about tumors is stored under this top-level header
  1) **<number>:** The second level enumerates the synchronous tumors. In our database, no patient has had a second tumor, but this structure of the file allows us to include such patients in the future. The third-level headers are the same for each tumor.
  1) **location:** Anatomical location of the tumor ("oropharynx" for all patients)
  2) **subsite:** ICD-O-3 code associated with a tumor at the particular location according to the world health organization [10,11]
  3) **side:** Lateralization of the tumor. Can be “left” or “right” for tumors that have their center of mass clearly on the respective side of the mid-sagittal line and “central” for patients with a tumor on the mid-sagittal line.
  4) **central:** Whether the tumor is centralized or not.
  5) **extension:** True if part of the tumor extends over the mid-sagittal line
  6) **volume:** Volume of the tumor in cm^3^
  7) **stage_prefix:** Prefix modifier of the T-category. Can be “c” or “p”
  8) **t_stage:** T-category of the tumor, according to TNM staging

  3) **<diagnostic modality>:** Each recorded diagnostic modality is indicated by its own top-level header. In this file FNA, CT, MRI, PET, pathology and pCT (planning CT) are provided
  1) **info:**
   1) **date:** Day on which a diagnosis with the respective modality was performed
  2) **ipsi:** All findings of involved lymph nodes on the ipsilateral side of the patient's neck
  1) **<LNL>:** One column is provided for each recorded lymph node level. For each level true indicates at least one finding diagnosed as malignant lymph node in the respective LNL, false means no malignant lymph node has been found and an empty field indicates that no diagnosis is available for this LNL according to the respective diagnostic modality. <LNL> can be I, Ia, Ib, II, IIa, IIb, III, IV, V, VI, VII, VIII, IX, X.
  3) **contra:** Same as 3.ii but for the contralateral side of the patient's neck
  1) **<LNL>:** same as under 3.ii.a

### 3.1 Cohort Characteristics

**Fig. 1:**
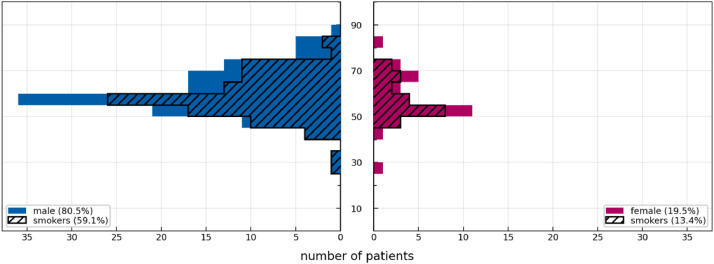
Histograms over the age of all men (blue, left) and women (red, right) in the cohort. The hatched histogram in each subplot represents the smokers.

Fig. 1 displays basic demographics of the cohort: 80.5% of all patients were male, 19.5% female. The median age was 58 years among men and 54 years among women. Tumors were HPV associated in 25.0% of men and in 34.4% of women.

**Fig. 2:**
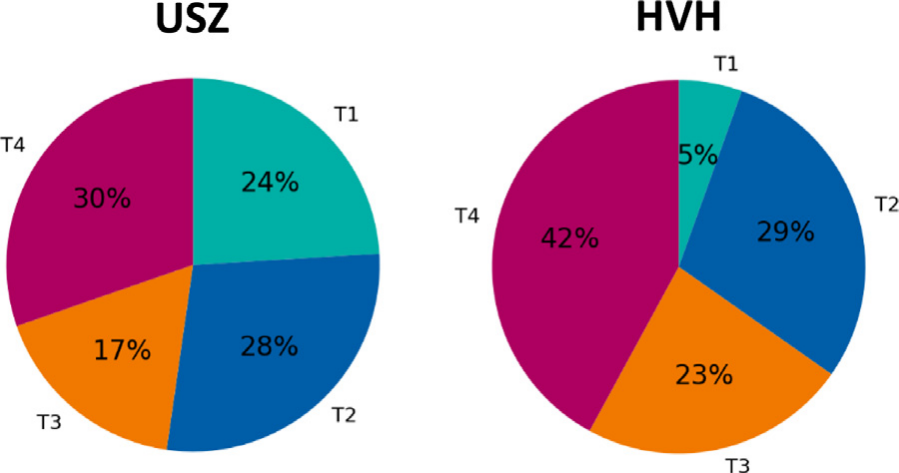
Comparison of distributions over T-categories in the original USZ dataset (left) and the newly added HVH cohort (right).

Fig. 2 shows the relative distribution of the different T-categories in the USZ and HVH dataset. Noteworthy is the small portion of T1 patients (HVH: 5%, USZ: 24%) and large portion of T4 patients (HVH: 42%, USZ: 30%).

**Fig. 3:**
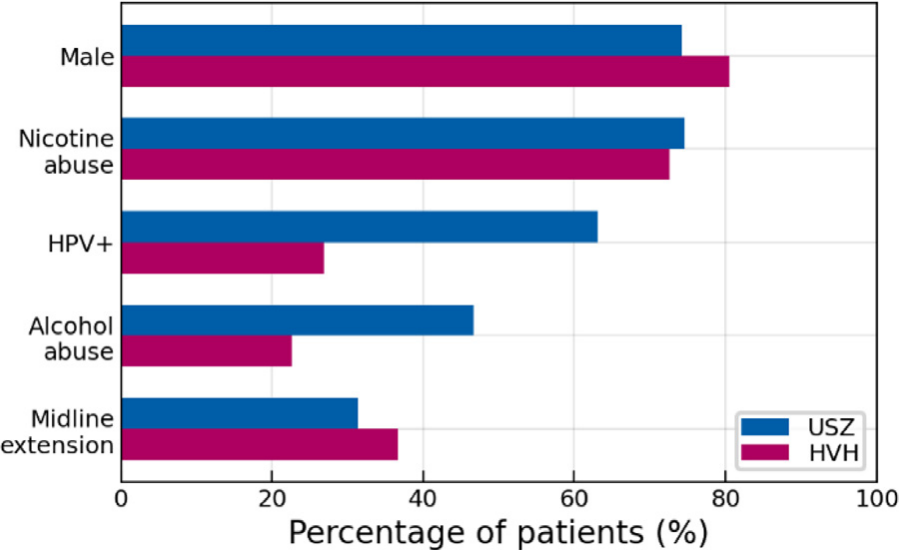
Comparing the prevalence of five clinicopathological factors between the original USZ dataset and the newly added HVH data.

The distribution of sex, the prevalence of smokers as well as the frequency of tumors crossing the mid-sagittal plane of the new HVH data is very close to what was observed at the USZ (Fig. 3). However, substantially fewer patients presented as HPV positive (HVH: 26%, USZ: 63%). Also, alcohol abuse was notably less prevalent at HVH (23%) compared to the USZ (47%).

**Fig. 4:**
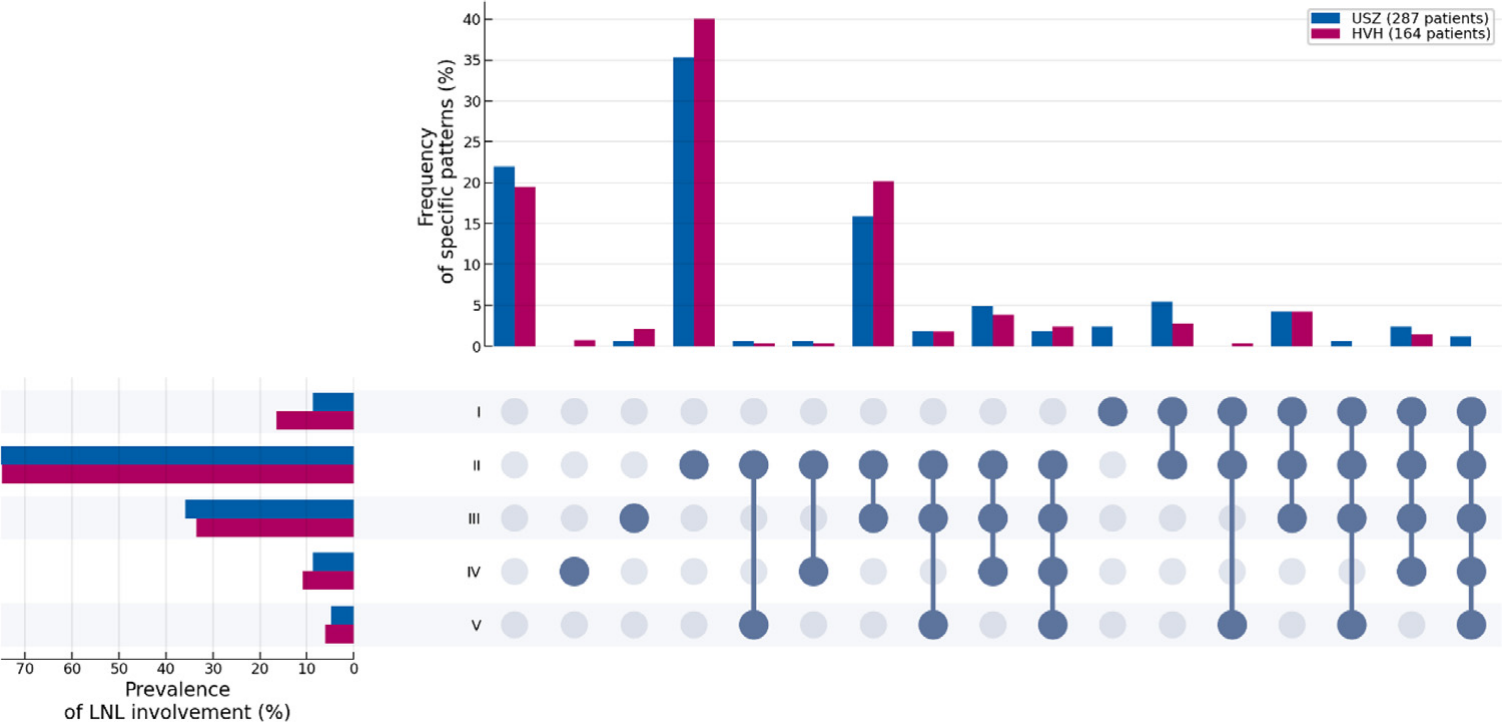
An “upsetplot” comparing the ipsilateral involvement patterns of the first reported dataset from the USZ and the here newly added data from the HVH. It shows both the prevalence of any ipsilateral LNL's involvement (horizontal bar plot, left), as well as how frequent any specific pattern of involvement was (vertical bar plot, top). The matrix of connected blue dots specifies these patterns: E.g, the first column represents the pattern where no LNL showed involvement (i.e, N0), while the ninth column – showing blue dots at LNL II, III, and IV – stands for the involvement of the LNLs from II to IV.

Lastly, we compare the ipsilateral involvement patterns of the USZ patients to the added dataset from the HVH. In Fig. 4 we present a so-called upsetplot, displaying both the overall prevalence of any LNL's involvement (histogram on the left) and the frequency of specific co-involvements of multiple LNLs. For example, the figure shows that 20% of HVH patients had clinical involvement of only the LNLs II and III, compared to 16% at the USZ.

## 4 Experimental Design, Materials and Methods

The dataset includes patients that were newly diagnosed with oropharyngeal squamous cell carcinoma and subsequently underwent treatment at Vall d'Hebron University Hospital (HVH) in the years from 2004 to 2021. The resulting table of 164 records contains patients treated with definitive radiotherapy, postoperative radiotherapy or surgery alone. Exclusion criteria include recurrent tumors or prior treatment of the neck. The data was collected by an experienced radiation oncologist who reviewed radiology and pathology reports as well as diagnostic images.

### 4.1 Criteria for clinical lymph node involvement

Whether a lymph node was considered malignant followed the description in Biau et al [6], which we repeat here:

• CT and MRI: Lymph nodes were considered involved when the smallest transverse diameter was larger than 1 cm. Further, nodes with central necrosis and/or loss of fatty hilum were also counted as involved.
• PET-CT: The above criteria as well as an SUV uptake of 2.5 or more were used to define positive involvement of a lymph node. Further, nodes not fulfilling the above CT/MRI criteria but showing an SUV uptake of more than 2.5 were also considered positive if there were three or more subcentimetric nodes grouped, or if the FDG uptake was intense.

### 4.2 Pathology after neck dissection

For most patients that received a neck dissection, resected lymph node levels were sent to the pathologist separately, who then reported the number of removed versus the number of positive lymph nodes per level. Occasionally, en bloc resection of neighboring node levels was performed, and the surgeon assigned the lymph node level based on clinical presentation. In cases where several nodal metastases formed a conglomerate or the involved nodes were not reported by level, the total number of removed and involved nodes in the en bloc resected region are reported. In most cases, the pathologist reported the largest metastatic node and the presence or absence of extracapsular extension.

### 4.3 Primary tumor characteristics

When extending over the mid-sagittal line, tumors were labelled to originate in the left/right side of the neck based on the location of the main primary mass. For central tumors lying on the midline, we defined the ipsilateral side to be that with more severe lymphatic involvement. All tumors were assigned to a specific subsite of oropharyngeal cancer (ICD-O-3 code).

## Limitations

As the original dataset from USZ, this updated dataset from the HVH only contains clinically assessed lymph node involvement for most patients. Pathologically assessed lymph node involvement is available for only 10 patients. Thus, lymph node involvement may be overestimated due to false-positive clinical diagnoses or underestimated due to false-negative clinical diagnoses.

The low number of T1 tumor patients and high percentage of T4 patients in the HVH dataset indicates a potential patient selection bias. The difference may in part be explained by a lower percentage of HPV associated tumors in the HVH dataset. Another explanation may be regional referral patterns: Early T-category cases may be treated in smaller local centers, while more advanced cases get referred to the HVH.

## Ethics Statement

The research has been carried out in accordance with The Declaration of Helsinki. The retrospective data collection was authorized by the HVH ethics committee (PR(AG)549/2024).

## Credit Author Statement

Sergi Benavente: Investigation, Data curation, Writing – review & editing

Roman Ludwig: Software, Visualization, Writing – original draft

Panagiotis Balermpas: Conceptualization, Writing – review & editing

Jan Unkelbach: Validation, Project administration, Writing – review & editing

## Data Availability

zenodo2025 HVH Oropharynx (Original data).
